# Rainfall seasonality predicts the germination behavior of a tropical dry‐forest vine

**DOI:** 10.1002/ece3.5108

**Published:** 2019-04-04

**Authors:** Adriana A. Martins, Øystein H. Opedal, William Scott Armbruster, Christophe Pélabon

**Affiliations:** ^1^ Department of Biology, Centre for Biodiversity Dynamics Norwegian University of Science and Technology, NTNU Trondheim Norway; ^2^ Faculty of Biological and Environmental Sciences, Research Centre for Ecological Change University of Helsinki Helsinki Finland; ^3^ School of Biological Sciences University of Portsmouth Portsmouth UK; ^4^ Institute of Arctic Biology University of Alaska Fairbanks Alaska

**Keywords:** after‐ripening, *Dalechampia scandens*, delayed germination, germination behavior, local adaptation, seasonal environments, seed dormancy, seed size, tropical dry forest

## Abstract

Seed dormancy is considered to be an adaptive strategy in seasonal and/or unpredictable environments because it prevents germination during climatically favorable periods that are too short for seedling establishment. Tropical dry forests are seasonal environments where seed dormancy may play an important role in plant resilience and resistance to changing precipitation patterns. We studied the germination behavior of seeds from six populations of the Neotropical vine *Dalechampia scandens* (Euphorbiaceae) originating from environments of contrasting rainfall seasonality. Seeds produced by second greenhouse‐generation plants were measured and exposed to a favorable wet environment at different time intervals after capsule dehiscence and seed dispersal. We recorded the success and the timing of germination. All populations produced at least some dormant seeds, but seeds of populations originating from more seasonal environments required longer periods of after‐ripening before germinating. Within populations, larger seeds tended to require longer after‐ripening periods than did smaller seeds. These results indicate among‐population genetic differences in germination behavior and suggest that these populations are adapted to local environmental conditions. They also suggest that seed size may influence germination timing within populations. Ongoing changes in seasonality patterns in tropical dry forests may impose strong selection on these traits.

## 1 INTRODUCTION

Successful plant establishment in seasonal environments requires accurate timing of germination to match favorable environmental conditions. The timing of germination is often controlled by seed dormancy (sensu lato), defined as the temporary inability of viable seeds to germinate during some period of conditions favorable for germination (Baskin & Baskin, 2014; Finch‐Savage & Leubner‐Metzger, 2006; Vleeshouwers, Bouwmeester, & Karssen, 1995). Because of its effect on seedling establishment, germination behavior is expected to be subject to strong selection and to exhibit adaptation to local environmental conditions (Donohue, Rubio de Casas, Burghardt, Kovach, & Willis, 2010). Local adaptation in germination behavior is supported by its extensive variation within and among species and by the covariation between germination behavior and several biotic and abiotic factors (e.g., Donohue et al., 2010; Meyer, Kitchen, & Carlson, 1995; Rubio de Casas et al., 2017; Simons, 2014; Torres‐Martinez, Weldy, Levy, & Emery, 2017; Venable, 2007; Wagmann et al., 2012).

Optimal timing of seed germination depends on seeds detecting reliable environmental cues that indicate the onset of the favorable growing season. For example, seed germination in some alpine plants requires exposure to low winter temperatures followed by extended periods of warm weather, indicating the onset of spring (Schwienbacher, Navarro‐Cano, Neuner, & Erschbamer, 2011). However, if conditions are only ephemerally favorable, such as during “false springs” (mild weather during winter), germination would likely result in seedling mortality. Environments characterized by such unpredictability are therefore expected to select for more complex patterns of seed dormancy or other fail‐safe mechanisms (Clauss & Venable, 2000; Gremer & Venable, 2014; Venable, 2007).

Patterns of dormancy may also be correlated with certain characteristics of the seeds. Among species, seed size often covaries with the presence or the duration of dormancy (Jurado & Flores, 2005; Norden et al., 2009; Rubio de Casas et al., 2017), and it is generally expected that larger seeds germinate more rapidly than smaller ones (Rees, 1996; Venable & Brown, 1988). This expectation is not always met, however, suggesting that other selective factors influence the relationship between seed size and dormancy (Norden et al., 2009). The relationships between germination behavior, seed size and seasonality, as well as among‐taxon variation in these relationships, are critical to understanding the causes of observed variation in germination behavior within and among individuals and populations.

Tropical dry forests are seasonal environments characterized by alternating favorable (wet) and unfavorable (dry) seasons for seedling establishment and plant growth, thus posing challenges to plants analogous to those faced by plants in temperate and polar ecosystems. In tropical forests with marked dry seasons, the absence of moisture is a limiting factor for seedling recruitment during the dry season, and germination usually matches the onset of the wet season (Escobar, Silveira, & Morellato, 2018; Frankie, Baker, & Opler, 1974; Garwood, 1983). Recent observations of changes in precipitation patterns in the tropics have led to concerns about the resistance and resilience of these highly threatened ecosystems (Allen et al., 2017; Feng, Porporato, & Rodriguez‐Iturbe, 2013), yet we lack knowledge of the ability of plant populations to adapt their germination behavior to changes in rainfall seasonality (Rubio de Casas et al., 2017).

**Figure 1:**
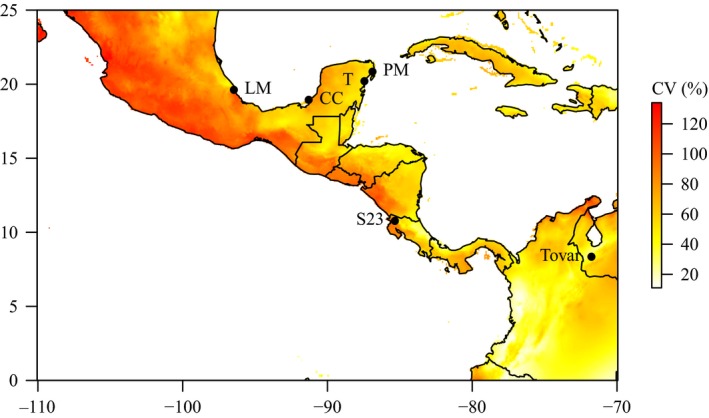
Map of *Dalechampia scandens* study populations, with colors indicating precipitation seasonality (CV of monthly precipitation in %). CC: Ciudad del Carmen; LM: La Mancha; PM: Puerto Morelos; S23: Rincón de la Vieja; T: Tulum

The euphorb vine *Dalechampia scandens* provides an excellent system for assessing how tropical dry‐forest plants adapt to seasonality. *Dalechampia scandens* occurs in habitats ranging from weak to pronounced seasonality (Figure 1). Flowering takes place at the end of the wet season and during the transitional period between the wet and dry seasons (Armbruster & Herzig, 1984). Many seeds are therefore dispersed during this transitional period and are exposed to intermittent rainfall followed by an extended period of drought. Consequently, some level of seed dormancy may be adaptive by preventing germination before the onset of the next full wet season, avoiding high seedling mortality during the intervening dry season (Escobar et al., 2018; Garwood, 1983; Ramos, Diniz, Ooi, Borghetti, & Valls, 2017).

If rainfall seasonality in tropical dry forests selects for seed dormancy, we expect that newly dispersed seeds will not immediately germinate when exposed to wet (favorable) conditions, and that, across sites, the time required to break dormancy and to germinate increases with increasing environmental seasonality. In two greenhouse experiments, we exposed seeds from *D. scandens* populations (originating from regions with differing seasonalities) to wet conditions, after experimental “dry seasons” of varying length, and measured the proportion of seeds not germinating as an index of dormancy. To test whether the durations of seed dormancy in these populations covary with local seasonality, we looked for possible correlations between the time required to break dormancy and the degree of precipitation seasonality in the region occupied by each population. We also tested whether within‐ and among‐population variation in seed size affected the time required to break dormancy and germinate.

## 2 MATERIALS AND METHODS

### 2.1 Study species and populations

**Figure 2:**
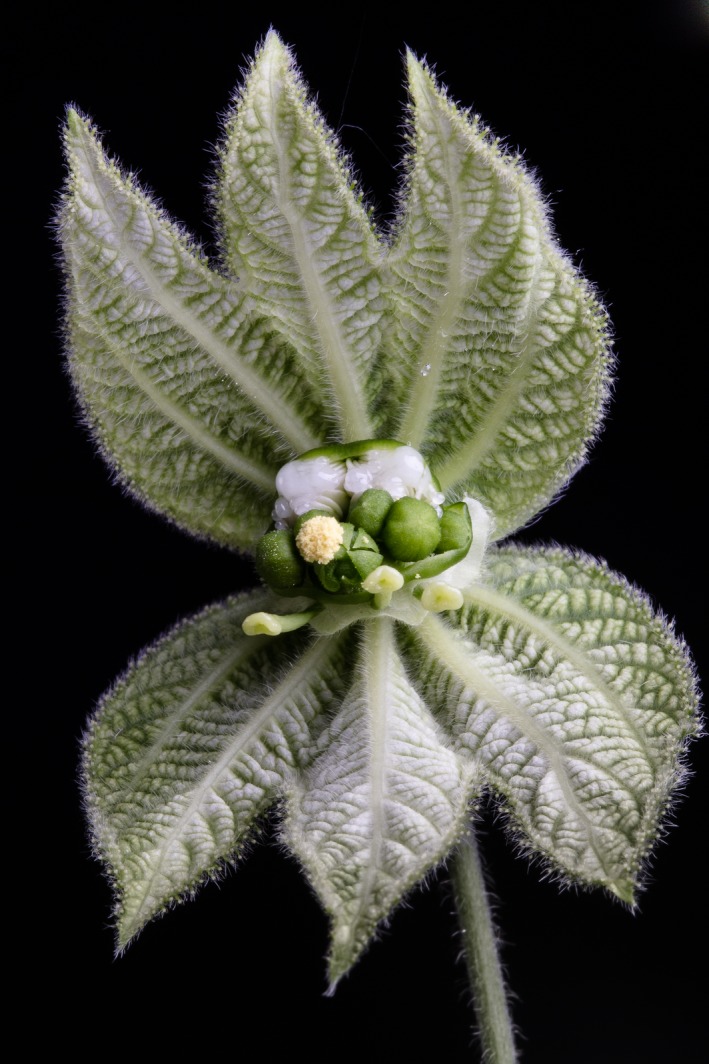
Pseudanthial blossom of *Dalechampia scandens *(Euphorbiaceae), a common vine occurring throughout the lowland Neotropics, from Mexico to Argentina. To assess the relationship between historical environmental conditions and seed dormancy, we studied the germination behavior of seeds from populations of this species originating from regions characterized by different degrees of seasonality. The photographed blossom is in the bisexual phase: visible are three receptive stigmas (borne on three pistillate flowers), one open male flower, and the resin gland (bearing white resin). (Photo by P.H. Olsen)

*Dalechampia scandens *L. (*s.l*.; Euphorbiaceae) is a species complex of perennial vines native to seasonally dry habitats in the Neotropics. Male and female unisexual flowers are aggregated into functionally bisexual inflorescences (Figure 2). A gland associated with the male subinflorescence secretes resin as a pollinator reward; this is collected by female apid (Apidae) and megachilid (Megachilidae) bees for use in nest construction. The pistillate subinflorescence comprises three female flowers, which can produce a maximum of nine seeds per inflorescence. Seeds disperse by explosive dehiscence of the capsules, normally four to 6 weeks after pollination. Seeds lack any apparent adaptations for secondary dispersal (Armbruster, 1982).

*Dalechampia scandens* is a pioneer species colonizing light gaps and other disturbed sites. Therefore, germination cues might include specific interactions between light, moisture, and other environmental factors. Because we were interested in variation in germination behavior in response to rainfall seasonality, we studied seed dormancy in six populations originating from regions characterized by different degrees of seasonality, that is, the contrast in precipitation between rainy and dry seasons (Figure 1, Table 1). The populations were chosen to represent the range of rainfall seasonality typically experienced by *D. scandens* in the study region. For example, the Tovar population (Merida, Venezuela) is characterized by relatively low seasonality in that it receives a low amount of rain throughout the year, while the highly seasonal La Mancha population (Veracruz, Mexico) receives nearly all rainfall during a 4‐ to 5‐month rainy season between June and October.

**Table 1:** Locations and summary statistics of study populations

Population	Coordinates	Altitude (m)	Rainfall seasonality (CV in %)	*n* (plants)	*n* (crosses)	*n* (seeds sown)	Seed size (*SD*)
First experiment							
Ciudad del Carmen	N 18°56′ W 91°18′	2	77	30	119	460	41.26 mg (4.24)
La Mancha	N 19°37′ W 96°28′	205	88	30	120	476	34.94 mg (2.28)
Puerto Morelos	N 20°51′ W 86°53′	7	51	30	120	475	46.90 mg (3.17)
Tulum	N 20°13′ W 87°26′	21	51	30	118	462	42.28 mg (2.92)
Second experiment							
Tovar	N 8°20′ W 71°46′	1,502	35	13	55	324	3.30 mm (0.13)
Rincon de la Vieja	N 10°46′ W 85°20′	774	58	13	38	202	3.62 mm (0.12)
Puerto Morelos	N 20°51′ W 86°53′	7	51	11	43	209	4.22 mm (0.11)
La Mancha	N 19°37′ W 96°28′	205	88	13	52	288	3.91 mm (0.17)

We analyzed data from two separate experiments in which we recorded germination of seeds exposed to wet environments after different durations of dry storage following fruit dehiscence. The presence of seed dormancy sensu lato is revealed by the temporary inability of viable seeds to germinate during some period of favorable conditions for germination (Finch‐Savage & Leubner‐Metzger, 2006; Vleeshouwers et al., 1995). Different functional classes of dormancy are recognized, depending on the proximal mechanism preventing germination (Baskin & Baskin, 2014,2004). Because we were primarily interested in comparing the relative duration of seed dormancy across the study populations regardless of the mechanisms involved, we chose to measure dormancy in the different populations as the duration of storage under dry conditions necessary to yield 50% germination when seeds were exposed to wet conditions. After‐ripening of seeds following dispersal is a common mechanism of dormancy break (Baskin & Baskin, 2014; Finch‐Savage & Leubner‐Metzger, 2006), and we thus assumed that our measure is closely associated to the timing of dormancy break during after‐ripening of seeds.

Both experiments were performed in a greenhouse with a 13:11‐light/dark regime and a temperature of 25°C during the day and 23°C at night. Because the maternal plants used in both experiments belonged to the second or later greenhouse generations, among‐population differences in germination behavior are presumed to be the result of genetic differences.

### 2.2 Experiment 1

In the first experiment, manual within‐population crosses were made among 30 individuals (four crosses per individual, total *n* = 120 crosses per population) from each of four populations (Table 1) over a 2.5‐month period (8 January–25 March) in 2014. We collected the seeds following explosive dehiscence of capsules 4–6 weeks after pollination, recorded the date of seed dispersal, and weighed the seeds on a precision balance (0.1 mg precision). Seeds were stored at room temperature in paper envelopes kept under dry conditions until sowing, during which after‐ripening presumably occurred. We sowed two seeds per cross on top of wet potting soil on the 14th of May, and two additional seeds per cross on the 12th of August (two temporal blocks). Because seeds dispersed over a 2.5‐month period (14 February–7 May), this yielded a nearly continuous distribution of after‐ripening durations, that is, duration of storage in a dry environment between capsule dehiscence and watering (mean = 97.9 days, *SD* = 48.1 days, range = 7–179 days). We recorded seed germination 1 month after sowing. Seeds were scored as germinated when the seed coat was broken, exposing the radicle.

### 2.3 Experiment 2

Within‐population crosses were made among 11–13 plants from each of four populations (Table 1) over a 2‐month period in August and September 2017. Because populations differ in duration of fruit maturation, we performed the crosses at different times to synchronize capsule dehiscence as much as possible. We recorded the date of seed dispersal and stored the seeds in white tea bags for up to 7 days in a dry place in the greenhouse under the same light and temperature conditions as those encountered by the maternal plants.

Each week during the period of fruit dehiscence, we initiated an experimental block (four blocks in total) comprising seeds matured during the preceding 7 days. From each seed set, we selected six healthy‐looking seeds and sowed them at random positions in six germination trays on top of dry regular sphagnum‐mixture potting soil. Aborted seeds with gray seed coats were discarded. We sowed all seeds at once without storing them in paper envelopes. This differed from the first experiment and aimed at controlling for a possible confounding effect of light on germination timing. Thus, in this experiment, seeds during after‐ripening were exposed to full greenhouse light with the same L:D regime as noted above.

Each tray was assigned to one watering treatment (four trays per treatment), and each seed set (seeds from a single blossom) was represented once in each treatment. Prior to sowing, we measured the diameter of each seed using digital callipers (0.01 mm precision). Each sowing tray with 45 cells was placed into a larger tray to which water could be added. All trays were placed on two tables in a single room in the greenhouse with similar temperature and light conditions as described above. The first tray (watering treatment *t*_0_) received water immediately after sowing and was maintained moist for the duration of the experiment. Subsequent trays received water 1, 2, 4, 8, and 16 weeks after the initiation of each block and were maintained moist. These were identified as watering treatments *t*_1_–*t*_16_, respectively. Each cell containing a seed was labeled with the identity of the seed.

We monitored the trays daily to record the number of days from watering to germination of each seed. Seeds were scored as germinated using the same criterion as in experiment 1. The experiment was terminated on 1 March 2018, at which time no new germination events had been observed for 2 weeks.

### 2.4 Statistical analyses

Despite slightly different designs, data from both experiments could be analyzed with statistical models containing the same biologically relevant parameters. We first assessed population differences in germination behavior for each experiment separately. To do so, we fitted generalized linear mixed‐effects models with binomial error distribution and logit link function to the data on germination success. Sowing tray (block), maternal identity, and blossom identity nested within maternal identity were treated as random effects, and the linear predictor of the models took the form ~*Population *+ *Duration of after‐ripening × Population *+ *Seed size × Population*. Seed size was measured as seed mass in the first experiment and seed diameter in the second experiment. In these models, duration of after‐ripening (i.e., the time from seed dispersal to watering) was treated as continuous for both experiments. Seed size was population‐mean centered (=observation–population mean) to compare the effect of after‐ripening duration on the probability of germination at the average seed size in each population.

As a measure of the relative duration of dormancy in each population, we used the parameter estimates from the models above to compute the duration of after‐ripening (exposure time to dry conditions prior to watering) necessary to yield 50% seed germination. We calculated *T*_50_ as *T*_50_ = −α/β_time_, where α is the intercept and β_time_ is the regression slope of the probability of seed germination on the duration of after‐ripening estimated for each population from the models above. Similarly, we assessed the effect of seed size on *T*_50_ by solving the logistic equation, yielding *T*_50_ = −(α + xβ_seed_)/β_time_, where *x* is seed size and β_seed _is the regression slope for seed size. Standard errors and 95% confidence intervals were obtained from 10,000 parametric bootstrap estimates drawn from the sampling distributions of the model parameters.

In the second experiment, we also evaluated the effect of seed size on the probability of germination within each watering treatment. We fitted separate generalized linear mixed‐effects model with binomial error distribution for each population. In these models, treatment was treated as a categorical variable and the treatments with no germinations were excluded from the analysis. We modeled the probability of germination as a function of watering treatment, seed size, and the treatment × seed size interaction (fixed effects) and included sowing tray (block), maternal identity, and blossom identity nested within maternal identity as random effects.

We also evaluated whether the time from watering to germination depended on the duration of after‐ripening or seed size in the second experiment. We modeled the time to germination (log‐transformed) as a function of population, watering treatment (duration of after‐ripening), and seed size (fixed effects) and included sowing tray (block), maternal identity, and blossom identity nested within maternal identity as random effects.

Finally, we analyzed the relationship between the population‐specific duration of seed dormancy estimated by *T*_50_ and the climatic conditions experienced by each population in its natural environment. As a measure of rainfall seasonality, we used the coefficient of variation (CV) of monthly precipitation averages for the period 1960–1990 extracted from WorldClim (Hijmans, Cameron, Parra, Jones, & Jarvis, 2005). Although alternative measures of rainfall seasonality and predictability are available (see e.g., Feng et al., 2013), we chose to analyze a single simple measure to avoid problems associated with multiple hypothesis testing based on *n* = 6 populations studied. All analyses were performed using R 3.5.0 (R Core Team, 2018).

## 3 RESULTS

### 3.1 Patterns of seed dormancy

**Figure 3:**
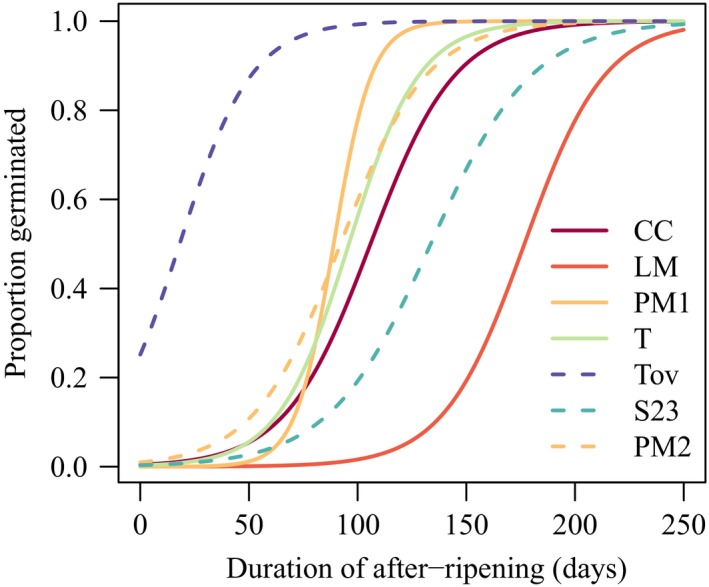
Estimated proportion of seeds germinating as a function of after‐ripening time, that is, the time from seed dispersal to watering. Solid lines are from the first experiment, and dashed lines are from the second experiment. Lines are extrapolated beyond the experimental periods to illustrate the full shape of the response curves (see Supporting Information Figures S2 and S3 for lines fitted to the data from each experiment)

In both experiments, the proportion of seeds germinating increased with longer exposure to dry conditions prior to watering, suggesting a gradual release from dormancy through after‐ripening of seeds (Figure 3, Supporting Information Table S1). This observation places *D. scandens* in the common class of nondeep physiological dormancy (PD sensu Baskin & Baskin, 2004).

In the first experiment, the three populations from the Yucatán peninsula (Ciudad del Carmen, Puerto Morelos, and Tulum) exhibited similar germination behavior, with 50% germination obtained after ca. 100 days of after‐ripening (Figure 3, Table 2). The population from La Mancha required longer after‐ripening to initiate germination, reaching 50% germination after nearly 180 days (Figure 3, Table 2).

**Table 2:** Parameter estimates ± *SE* for the germination models for the first and second experiments

Population	Intercept (log odds)	Time effect (log odds day^−1^)	Seed‐size effect	*T*_50_ (days)
First experiment				
Ciudad del Carmen	−5.40 ± 0.65	0.051 ± 0.006	−0.23 ± 0.06 log odds mg^−1^	105.94 ± 18.46
La Mancha	−9.46 ± 1.53	0.053 ± 0.010	−0.10 ± 0.10 log odds mg^−1^	176.72 ± 52.28
Puerto Morelos	−10.13 ± 1.19	0.114 ± 0.013	−0.18 ± 0.09 log odds mg^−1^	88.98 ± 15.46
Tulum	−5.90 ± 0.68	0.061 ± 0.006	−0.23 ± 0.08 log odds mg^−1^	96.00 ± 15.26
Second experiment				
Tovar	−1.09 ± 0.26	0.060 ± 0.009	−3.36 ± 1.40 log odds mm^−1^	18.14 ± 5.21
Rincon de la Vieja	−5.71 ± 0.96	0.043 ± 0.009	−8.59 ± 2.80 log odds mm^−1^	133.66 ± 45.31
Puerto Morelos	−4.62 ± 0.62	0.050 ± 0.007	0.01 ± 2.90 log odds mm^−1^	92.13 ± 19.22

In the second experiment, seeds from the Tovar population germinated in all watering treatments, and the proportion of germinating seeds reached 50% after <3 weeks of after‐ripening prior to watering (*T*_50_ = 18.14 ± 5.21 days). Furthermore, the time from watering to germination decreased strongly in later treatments (Supporting Information Figure S1, Table S3). Seeds from the Puerto Morelos population started germinating after 8 weeks of after‐ripening, reaching 50% after ca. 13 weeks (*T*_50_ = 92.15 ± 19.22 days). This result is nearly identical to that obtained in the first experiment with the same population, suggesting that the results of the two experiments can be directly compared and combined despite the differences in seed storage during after‐ripening. Seeds from the Rincon de la Vieja population did not reach 50% germination within the experimental period (*T*_50_ = 133.66 ± 45.31 days), and only a single seed from the La Mancha population germinated in the second experiment. Again, this confirms the results for the latter population obtained in the first experiment (Figure 3).

### 3.2 Effects of seed size on germination rate and timing

In both experiments, smaller seeds germinated more rapidly than did larger ones, as indicated by the negative effect of seed size on the probability of germination after a certain duration of after‐ripening in most populations (Table 2). In the first experiment, the seed‐size effect was very similar in the three populations from the Yucatán peninsula (Ciudad del Carmen, Puerto Morelos, Tulum). An increase in the seed mass by one standard deviation increased *T*_50_ by between 5.0 days (Puerto Morelos) and 18.8 days (Ciudad del Carmen, Figure 3). We did not detect an effect of seed mass on the timing of germination in the La Mancha population.

**Figure 4:**
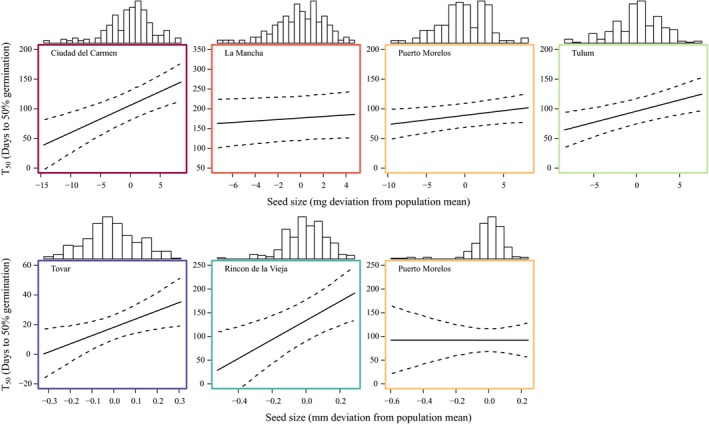
Effects of seed size on *T*_50_, the number of days of after‐ripening necessary to yield 50% germination, in the first (top row) and second (lower row) experiments. The solid lines are given by *T*_50_ = −(α + xβ_seed_)/β_time_ and are drawn over the range of seed sizes (*x*) in each population, as indicated by the histograms above each panel

**Figure 5:**
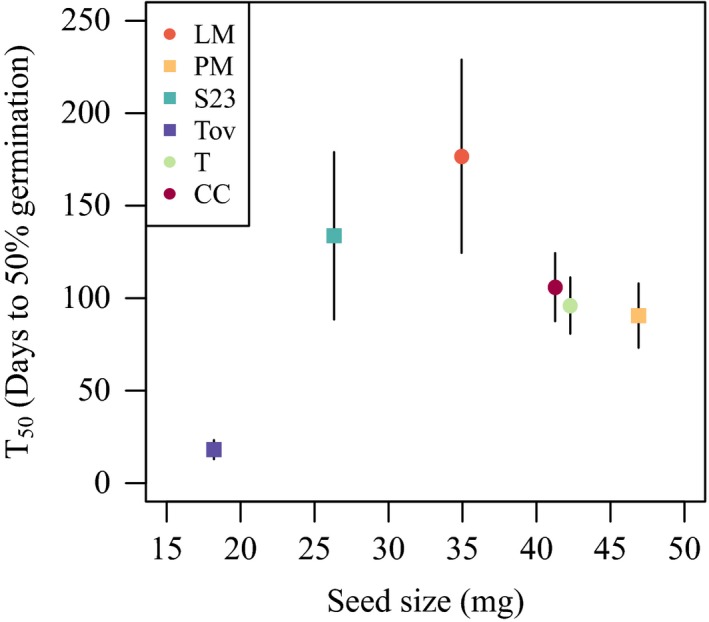
Among‐population relationship between mean *T*_50_, the number of days of after‐ripening necessary to yield 50% germination, and seed size (measured as seed mass in mg). Error bars indicate standard errors

In the second experiment, we also detected an apparent effect of seed size on the timing of germination in the Tovar and Rincon de La Vieja populations (Table 2). An increase in the seed size by one standard deviation increased *T*_50_ by 7.4 days and 21.8 days for Tovar and Rincon de la Vieja, respectively (Figure 4). In contrast to the first experiment, we detected no overall seed‐size effect in the Puerto Morelos population. However, the expected effect was detected when restricting the analysis to the final treatment with the greatest number of seeds germinating (Supporting Information Table S2). Seed size did not detectably affect the time from watering to germination (Supporting Information Table S3). Among populations, there was no systematic relationship between mean seed size and the number of days of after‐ripening necessary to yield 50% germination (Figure 5).

### 3.3 Dormancy versus rainfall seasonality

**Figure 6:**
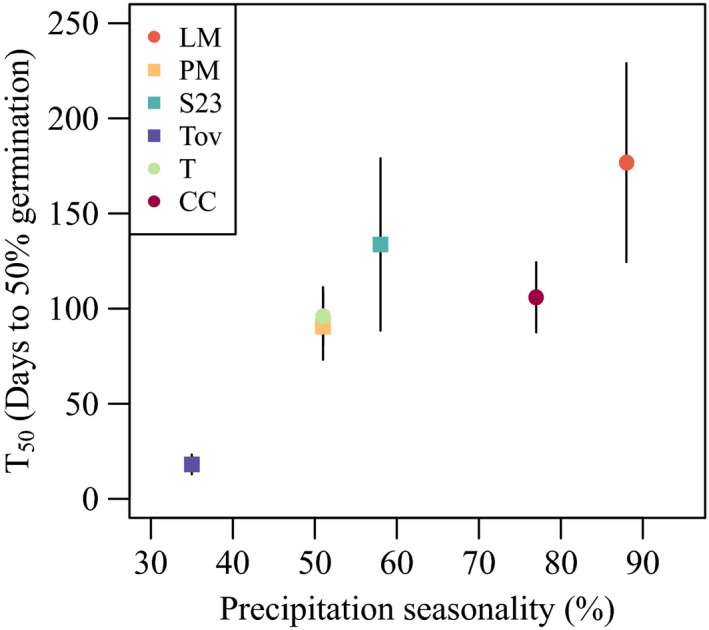
Among‐population relationship between mean *T*_50_, the number of days of after‐ripening necessary to yield 50% germination, and precipitation seasonality. Error bars indicate standard errors

Populations from more seasonal environments required longer after‐ripening before reaching 50% germination after watering (Figure 6). The Tovar population differed strongly from the other populations, while differences among the remaining populations were more subtle.

## 4 DISCUSSION

Seed dormancy is considered to be an adaptive strategy in seasonal and unpredictable environments because it prevents germination during periods that are only ephemerally favorable for seedling growth and establishment (e.g., Torres‐Martinez et al., 2017; Venable, 2007; Vleeshouwers et al., 1995). In the current experiment, we showed that, while all populations of *D. scandens* produced at least some dormant seeds, they differed in the duration of after‐ripening (pre‐watering period) necessary to initiate germination after watering. Because the seeds used in these experiments were produced in a common environment from plants of the second or later greenhouse generation, these differences most likely reflect genetic differentiation among populations in the duration of dormancy (*s.l*.). Furthermore, the positive correlation between the duration of dormancy and the seasonality of the environment experienced by each population is consistent with the hypothesis of locally adapted germination behavior, although this conclusion must be taken as provisional and awaits experimental demonstration.

In her extensive work on the germination behavior of plants in a seasonal moist tropical forest in Panama, Garwood (1983) classified species' regeneration strategies into three distinct syndromes (“delayed‐rainy,” “intermediate‐dry,” and “rapid‐rainy”), according to the time required for germination and the seasonal timing of seed dispersal. *Dalechampia scandens* fits the “delayed‐rainy” syndrome, characterized by seed dispersal in the late‐wet/early‐dry season, combined with dormancy. This appears to be a common strategy among tropical plants dispersing their seeds during the late rainy season (Escobar et al., 2018; Ramos et al., 2017; Sautu, Baskin, Baskin, Deago, & Condit, 2007; Silveira, Ribeiro, Oliveira, Fernandes, & Lemos‐Filho, 2011). The most straightforward interpretation of this strategy is that seed dormancy evolves as a mechanism to avoid germination following intermittent rains during the transition between the wet and dry seasons, therefore ensuring germination at the onset of the next wet season.

The adaptive interpretation of seed dormancy in tropical plants implicitly assumes that seeds can survive exposure to wet conditions for some time during the dormant period. In the second experiment, some seeds sown in the first watering treatments were maintained in wet environments for up to 5 months, yet failed to germinate. Failure to germinate might have been due to extended dormancy of seeds exposed to moist environments, or to the death of the seed as a result of fungal infection and/or rotting during prolonged exposure to high moisture. We did not perform seed viability tests, but we manually broke the seed coat of a sample of seeds at the end of the second experiment, and in no case did the seeds show signs of rotting. We therefore tentatively conclude that *D. scandens* seeds can survive for extended periods in wet conditions during the dormant phase. The occurrence of seed banks in tropical dry forests is poorly known (Skoglund, 1992), but our results suggest that *D. scandens* exhibits at least a short‐term seed bank. Annual dormancy cycles, as responses to the sequence of environmental conditions affecting the seed bank, are well known in temperate regions (Bouwmeester & Karssen, 1993; Vleeshouwers et al., 1995). Similar data are, however, lacking for tropical plants. Some work suggests that seeds of tropical plants can also survive for extended periods in the soil (Vazquez‐Yanes & Orozco‐Segovia, 1993), but we consider it likely that most *D. scandens* seeds germinate at the onset of their first full wet season, at least if exposed to sun.

Our experimental design allowed us to assess genetic differences in dormancy in a common environment. The duration of dormancy under natural conditions might, however, differ from what we observed in the greenhouse due to maternal environmental effects (Donohue, 2009; Postma & Ågren, 2015). Garwood (1983) observed differences in the time to germination when seeds of the same species were collected at different times of the year, and preliminary work with field‐collected *D. scandens* seeds suggests similar patterns (Ø. H. Opedal, unpublished results). For example, the benign conditions experienced by our experimental plants (constant water availability) may be a cue suggesting that the dry season is still to come, and the seeds produced may be dormant for a longer time than seeds produced under drier conditions at the beginning of the dry season.

In all but one population, we detected negative relationships between seed size and the fixed‐time germination probability within populations, after controlling for the duration of after‐ripening (Table 2). In other words, smaller seeds were more likely to germinate after a given period of after‐ripening, yielding a positive relationship between seed size and *T*_50_ (Figure 4). Positive relationships between seed size and duration of dormancy have also been reported at the species level (Norden et al., 2009). Norden et al. (2009) suggested that this pattern results from morphological constraints associated with reduced time required either to mature seeds or to imbibe water as seeds become smaller. Importantly, while smaller seeds required shorter after‐ripening before germinating, larger seeds may perform better once they germinate (Moles & Westoby, 2004; Pélabon, Carlson, Hansen, & Armbruster, 2005). Furthermore, earlier germination may be selected against if it occurs in response to ephemerally favorable conditions and thus leads to seedling mortality (see Donohue et al., 2010 for a review on natural selection on germination timing). Among *D. scandens *populations, seed size did not detectably correlate with the duration of dormancy (Figure 5). Together, these observations suggest complex relationships between seed size, germination timing, and fitness.

Differences in population‐specific germination behavior were correlated with the within‐year variation in rainfall experienced historically by each population. Populations from less seasonal environments, particularly Tovar, required shorter duration of after‐ripening to initiate germination than did populations from more seasonal environments, particularly La Mancha. These observations are consistent with the hypothesized importance of seasonality as an environmental factor selecting on dormancy duration (Rubio de Casas et al., 2017). However, we cannot ascertain whether this correlation is causal. While the measure of seasonality used in this study (CV of mean monthly precipitation) apparently captures some component of environmental variation important for determining dormancy in *D. scandens*, it is unlikely to be the single proximal driver of seed dormancy. Indeed, selection on dormancy is presumably related to the probability of experiencing ephemeral favorable conditions, such as intermittent rainfalls during the transitional period between the wet and the dry seasons (Clauss & Venable, 2000), a characteristic of the environment that is not directly captured by the CV of monthly rainfall. In the vernal pool plant *Lasthenia fremontii *in California, for example, germination behavior varied predictably with historical variation in autumn precipitation (Torres‐Martinez et al., 2017). It is possible that more seasonal tropical environments are also highly variable among years, so that the probability of late rainfalls during the period of seed dispersal is greater in those environments.

Our results are potentially important in the light of recent and predicted changes in seasonal patterns of precipitation in the tropics (Allen et al., 2017; Feng et al., 2013). If the probability of rainfall during the dry season increases, we might expect strong selection on dormancy, especially in populations currently occupying more seasonal environments. Interestingly, the effect of seed size on germination behavior in *D. scandens* suggests that selection on germination timing could also lead to evolutionary shifts in seed size if the two traits are genetically correlated. Whether populations can respond to selection imposed by novel climatic conditions depends on the additive genetic variance in germination behavior within populations. Few studies have quantified the evolvability of germination traits. Simons and Johnston (2006) reported substantial additive genetic variance for germination time in *Lobelia inflata*, but even greater environmental variance. Such patterns are common for life‐history traits (Houle, 1992), but it is not entirely clear how this affects their evolutionary dynamics. Low additive genetic variance in seed size has been commonly reported (e.g., Schwaegerle & Levin, 1990; Pélabon, Albertsen, Falahati‐Anbaran, Wright, & Armbruster, 2015; Pélabon et al., 2016) and may constrain the evolution of germination behavior mediated by seed size.

## 5 CONCLUSION

Our study demonstrates that *D. scandens* populations have evolved degrees of seed dormancy consistent with the hypothesis of local adaptation to local climatic conditions. However, a complete demonstration of local adaptation will require field studies quantifying the fitness consequences of variation in germination timing, preferably over multiple seasons. One important question arising from our work concerns how exposure to moist conditions during the dormant period affects the timing of dormancy release and the subsequent viability of seeds. We suspect that the patterns observed in *D. scandens* will apply to many tropical dry‐forest species, and we hope that our results will motivate further studies of the germination behavior of tropical plants and how it relates to climatic patterns.

## CONFLICT OF INTEREST

None declared.

## AUTHOR CONTRIBUTIONS

ØHO and CP initiated the study. AAM, ØHO and CP designed the experiments. AAM and ØHO collected data, performed analyses, and wrote the first draft of the manuscript. All authors contributed substantially to revisions.

## Data Availability

Data supporting the results: Dryad Data Repository (Provisional https://doi.org/10.5061/dryad.fd4j10p)
