# Supplementary material for: Rainfall seasonality predicts the germination behavior of a tropical dry‐forest vine
Source: Ecol Evol. 2019 Apr 4;9(9):5196–205. doi: 10.1002/ece3.5108 (PMC6509399; doi:10.1002/ece3.5108)
Supplement: Supplementary file 1 [file ECE3-9-5196-s001.docx]

**Rainfall seasonality predicts the germination behaviour of a tropical dry-forest plant**

Adriana A. Martins^1,*^, Øystein H. Opedal^1,2^, W. Scott Armbruster^3,4^ and Christophe Pélabon^1^

**SUPPLEMENTARY MATERIALS**

Table S1. Model selection results for the germination models in the first and second experiments.

Table S2. Parameter estimates ± SE for effects of seed size for each treatment in the second experiment.

Table S3. Parameter estimates ± SE for the effect of after-ripening time and seed size on time from watering to germination in the second experiment.

Fig. S1. Cumulative seed germination as a function of after-ripening time in the second experiment. Lines and dots with different colours represent different treatments, and figure outline colours represent the different populations.

Fig. S2: Estimated proportion of seeds germinating as a function of after-ripening time (time from seed maturation to watering) in the first experiment. Abbreviations: CC = Ciudad del Carmen, LM = La Mancha, PM = Puerto Morelos, T = Tulum.

Fig. S3: Observed (dots) and estimated (lines) proportion of seeds germinating as a function of after-ripening time (time from seed maturation to watering) in the second experiment. Dots show the proportion germination in each watering treatment. Abbreviations: Tov = Tovar, S23 = Rincón de la Vieja, PM = Puerto Morelos.

| Table S1. Model selection results for the germination models in the first and second experiments. k is the number of parameters in the model, logLik is the log likelihood, and ΔAIC is the difference in AIC value from the highest ranked model. | | | |
| --- | --- | --- | --- |
| Model parameters | k | logLik | ΔAIC |
| *First experiment* |  |  |  |
| Pop + Pop × Treatment + Pop × Seed size | 16 | -432.4 | 0 |
| Pop + Treatment + Seed size | 10 | -455.8 | 34.79 |
| Pop | 8 | -511.2 | 141.65 |
| Intercept only | 5 | -521.3 | 155.77 |
| *Second experiment* |  |  |  |
| Pop + Pop × Treatment + Pop × Seed size | 13 | -242.6 | 0 |
| Pop + Treatment + Seed size | 9 | -246.9 | 0.72 |
| Pop | 7 | -363.5 | 229.87 |
| Intercept only | 5 | -370.2 | 239.39 |

| Table S2. Parameter estimates ± SE for effects of seed size for each treatment in the second experiment. Estimates are shown for treatments with more than one seed germinating. | | | |
| --- | --- | --- | --- |
| Population | Treatment | Intercept  (log odds) | Seed size effect  (log odds mm^-1^) |
| Tovar | t_0_ | 13.75 ± 13.05 | -4.99 ± 4.01 |
|  | t_1_ | 13.15 ± 9.38 | -4.27 ± 2.85 |
|  | t_2_ | 15.09 ± 9.17 | -4.53 ± 2.77 |
|  | t_4_ | 15.36 ± 10.14 | -4.28 ± 3.05 |
|  | t_8_ | -1.24 ± 11.79 | 1.15 ± 3.59 |
|  | t_16_ | 15.23 ± 17.87 | -3.65 ± 5.36 |
| Rincon de la Vieja | t_8_ | 56.54 ± 32.93 | -16.78 ± 9.47 |
|  | t_16_ | 26.00 ± 18.87 | -7.41 ± 5.24 |
| Puerto Morelos | t_8_ | -30.59 ± 25.21 | 7.03 ± 5.96 |
|  | t_16_ | 45.84 ± 31.38 | -10.64 ± 7.38 |

| Table S3. Parameter estimates ± SE for the effect of after-ripening time and seed size on time from watering to germination in the second experiment. | | | |
| --- | --- | --- | --- |
| Population | Intercept (log days) | Time effect (log days day^-1^) | Seed size effect (log days mm^-1^) |
| Tovar | 3.09 ± 0.08 | -0.0097 ± 0.0008 | 0.51 ± 0.31 |
| Rincon de la Vieja | 2.04 ± 0.41 | -0.0014 ± 0.0037 | -0.45 ± 0.86 |
| Puerto Morelos | 2.50 ± 0.29 | -0.0045 ± 0.0029 | -0.51 ± 0.90 |


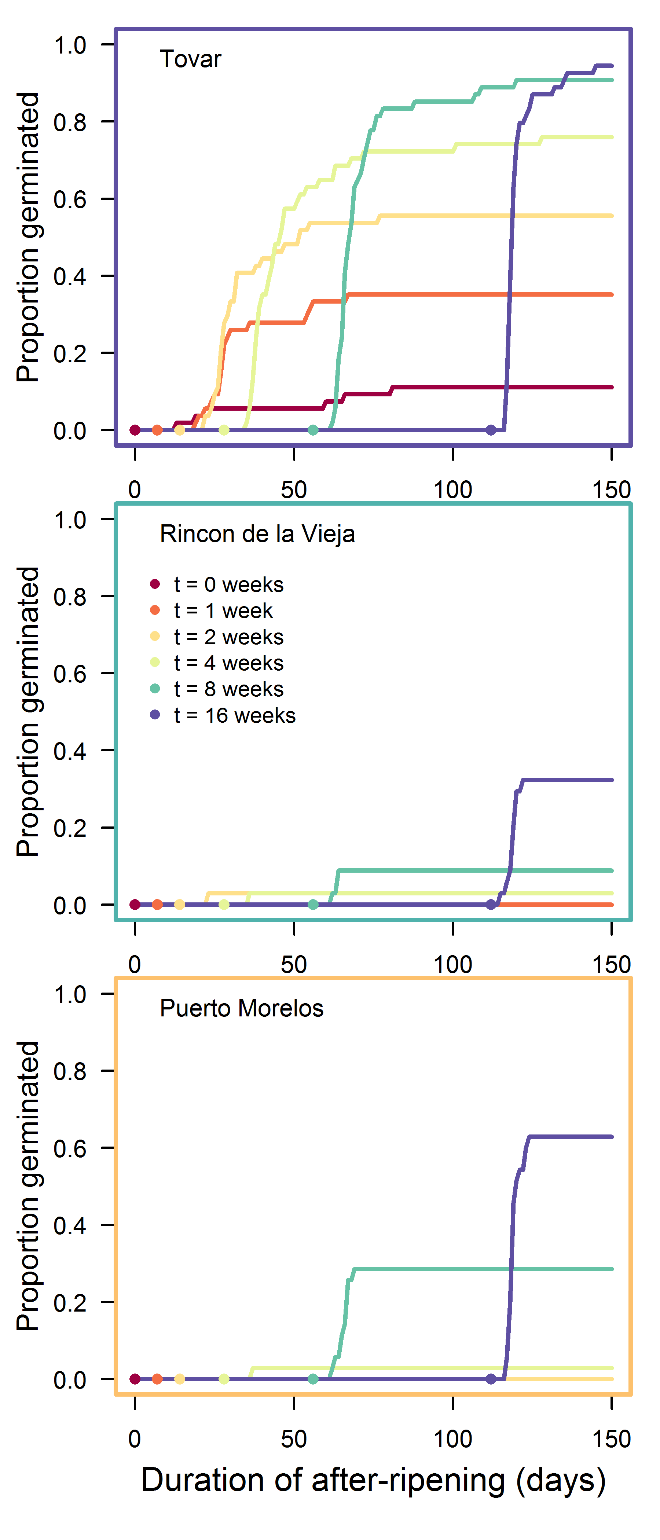


Fig. S1. Cumulative seed germination as a function of after-ripening time in the second experiment. Lines and dots with different colours represent different treatments, and figure outline colours represent the different populations.


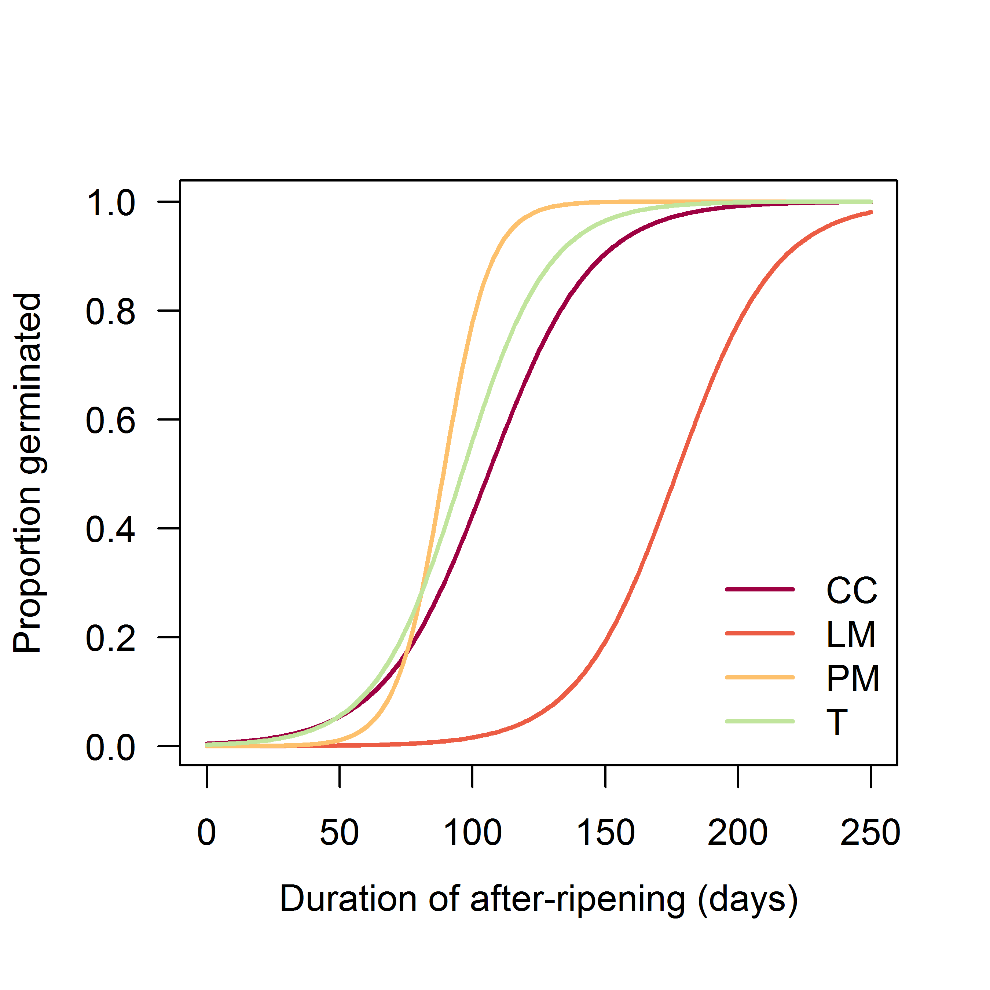


Fig. S2: Estimated proportion of seeds germinating as a function of after-ripening time (time from seed maturation to watering) in the first experiment. Abbreviations: CC = Ciudad del Carmen, LM = La Mancha, PM = Puerto Morelos, T = Tulum.


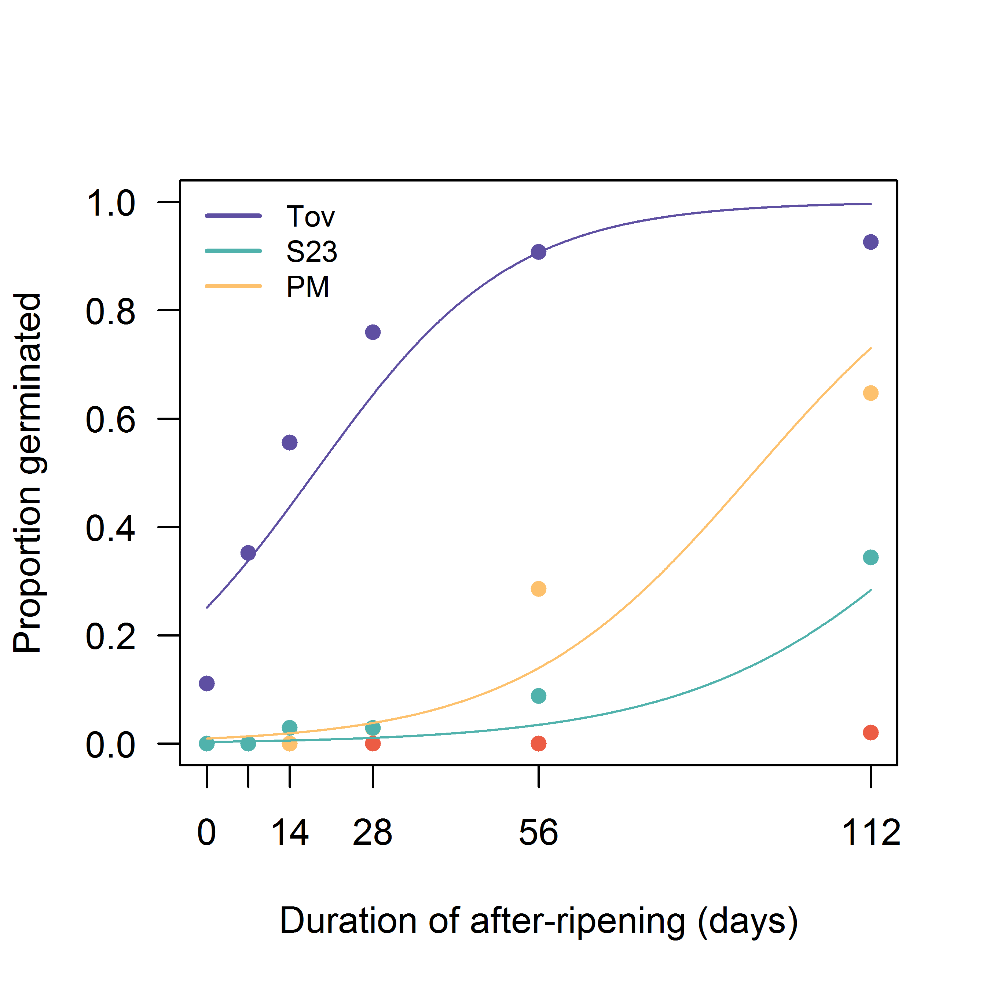


Fig. S3: Observed (dots) and estimated (lines) proportion of seeds germinating as a function of after-ripening time (time from seed maturation to watering) in the second experiment. Dots show the proportion germination in each watering treatment. Abbreviations: Tov = Tovar, S23 = Rincón de la Vieja, PM = Puerto Morelos.
